# Biopsy-proven acute tubulointerstitial nephritis in patients treated with immune checkpoint inhibitors: a pooled analysis of case reports

**DOI:** 10.3389/fonc.2023.1221135

**Published:** 2023-10-23

**Authors:** Pasquale Esposito, Annarita Bottini, Elvina Lecini, Francesca Cappadona, Michela Piaggio, Lucia Macciò, Carlo Genova, Francesca Viazzi

**Affiliations:** ^1^ Nephrology Unit, IRCCS Ospedale Policlinico San Martino, Genova, Italy; ^2^ Department of Internal Medicine, University of Genova, Genova, Italy; ^3^ UOC Clinica di Oncologia Medica, IRCCS Ospedale Policlinico San Martino, Genova, Italy

**Keywords:** immune checkpoint inhibitors, cancer, acute kidney injury, acute tubulointerstitial nephritis, pooled analysis, corticosteroids, renal biopsy, chronic kidney disease

## Abstract

**Introduction:**

Acute kidney injury (AKI) in cancer patients receiving immune checkpoint inhibitors (ICIs) may recognize multiple causes. Here, we reviewed cases of biopsy-proven acute tubulointerstitial nephritis (ATIN) to describe the clinical characteristics and outcomes of this condition.

**Method:**

We conducted a pooled analysis of clinical cases of ICI-related biopsy-proven ATIN up to 1 May 2022. We collected data on clinical characteristics, AKI, biopsy findings, laboratory examinations, and renal outcomes.

**Results:**

Eighty-five patients (61.4 ± 19 years, 56 male) were evaluated. Melanoma was the most prevalent diagnosis (51%), followed by non-small cell lung cancer (30%). ICI treatment consisted of PD-1, PDL-1 (nivolumab, pembrolizumab, atezolizumab), and CTLA-4 inhibitors (i) (ipilimumab) or combination PD-1i+CTLA4i. Renal toxicity developed after a median of four cycles of therapy. Fifty-one patients (65.5%) developed the most severe form of AKI- stage 3, including five patients requiring dialysis. All the 19 patients treated with dual ICI blockade developed AKI-stage 3, compared with 29 patients out of the 60 receiving a single agent (p<0.001). Most events were managed with corticosteroids associated with ICI withdrawal. In 15 patients ICI was restarted, but in six (40%) AKI recurred. Overall, 32 patients (40%) presented a complete renal recovery, which chance was inversely associated with dual ICI blockade (OR 0.15, 95CI 0.03-0.7, p=0.01).

**Conclusion:**

ICI-related ATIN may develop late after the therapy initiation, presenting as severe AKI, particularly in patients with dual ICI blockade. Although this complication may be partially reversible, concerns remain about the renal function sequelae and the possibility of restarting ICI treatment.

## Introduction

The systemic treatment with immune checkpoint inhibitors (ICIs) has revolutionized the management of multiple solid tumors, including melanoma and non-small cell lung cancer (NSCLC) (1). Notably, ICIs have been initially employed as single-agent regimens in advanced tumors; however, their use has been progressively extended to different settings, and novel combinations have emerged, including combinations of ICI and chemotherapy or combinations of multiple ICIs. Currently, the main immune checkpoints of therapeutic interest are the Programmed Death protein 1 (PD-1) and its ligand (PD-L1), as well as Cytotoxic T Lymphocyte Antigen 4 (CTLA-4) (2).

This treatment is not completely free from potential adverse events, which are strictly correlated with their mechanism of action (3). Indeed, disrupting the physiologic inhibitory effect of immune checkpoints might result in lymphocyte activation against multiple normal sites; such toxicities, collectively defined as immune-related adverse events (irAEs), may virtually involve any site, such as skin, gastroenteric tract, lungs, or kidneys (4).

Acute kidney injury (AKI) is a relatively uncommon irAE, but it has a relevant impact on involved patients (5). Indeed, AKI might translate into long-term renal dysfunction that might result in ineligibility to receive subsequent lines of treatment, such as chemotherapy, with a potential negative impact on patients’ outcomes from an oncological perspective (6). Notably, while clinical trials and published real-world data report immune-related renal toxicities, in most cases such events are only described as “creatinine increase” and renal biopsies are seldom performed (7, 8). Ideally, a renal biopsy might provide relevant data, both in terms of differential diagnosis and toxicity grading beyond the simple creatinine value, with a potential role in prognostic information (9). The most common histological finding in patients with ICI-related AKI is acute tubulointerstitial nephritis (ATIN), characterized by inflammation, infiltration of immune cells, and tubular damage (10). However, AKI in cancer patients is a multifaceted phenomenon that may recognize multiple causes, so the etiological definition is fundamental to address a proper clinical approach.

In this study, we reviewed case reports of biopsy-proven ATIN in patients treated with ICIs, to describe the clinical and laboratory characteristics and outcomes of this condition.

## Methods

### Search strategy, study selection, and data extraction

A literature search was conducted on PubMed for eligible studies published up to May 1, 2022, using the search terms [(Pembrolizumab OR Nivolumab OR Ipilimumab OR Atezolizumab OR Avelumab OR Durvalumab) AND (Nephritis)]. No filters were applied, and the text words could be in all fields of the articles. Data were independently extracted by two investigators (A.B. and E.L.) who carefully and manually scanned all the articles to identify cases of biopsy-proven ATIN A supervisor reviewed any discrepancies (P.E.). A total of 139 articles were identified by the search on PubMed, and 43 were eligible for analysis (7, 8, 11–51). The selection process was performed in two steps. In the first step, we analyzed articles, excluding those not presenting case reports/case series, not in English or not involving checkpoint inhibitors. Then, from this initial list, articles without renal biopsy, articles reporting other renal alterations, and those with insufficient clinical data were also excluded.

Of note, the list of papers excluded from the analysis also comprised the multicenter study by Gupta el. 2021 which evaluated more than 400 patients with ICI-related AKI (52). Indeed, although this cohort included 151 patients undergoing kidney biopsy (89% of them with ATIN), the authors did not provide detailed information to discern and characterize the clinical presentation and course of patients with biopsy-proven ATIN, with respect to patients with AKI from other etiologies.

The final list of eligible articles included 111 patients.

After the first article selection, as a second step, we analyzed individual patients, excluding 28 cases reporting only extra-renal toxicity, other renal alterations than ATIN, or cases without renal biopsy. Therefore, 83 patients were suitable for analysis, to whom we added two patients from our institution (who gave written informed consent to data collection), reaching a final number of 85 patients. (**Supplementary Figure 1**) The extracted data included patient demographics, comorbidities, home therapy, histology cancer, oncological therapy regimen, number of therapy cycles before renal toxicity, results of renal biopsy (in addition to ATIN, we recorded also the compresence of granulomas or acute tubular necrosis), baseline serum creatinine (sCr) and slope, clinical presentation (blood pressure, edema, hematuria), presence of other checkpoint inhibitor-induced toxicities, urinary analysis, ATIN treatment (dosage and duration), and decisions regarding restarting or interrupting ICIs therapy.

### Definitions

We defined and graded AKI according to the Kidney Disease: Improving Global Outcomes (KDIGO) Clinical Practice Guideline, based on changes in serum creatinine with respect to the pre-ICI value considered as baseline (53). We defined each stage as stages 1, 2, and 3.

These correspond to a serum creatinine increase of 1.5 to 1.9 times from the baseline value or a serum creatinine peak ≥ 0.3 mg/dL, 2 to 2.5 times, and 3 or more times their baseline creatinine or newly required renal replacement therapy (RRT). Urine output was not considered due to limited data availability.

Hematuria was defined as a red blood cell number of more than three per high-power field, and nephrotic proteinuria as proteinuria of more than 3.5 g/day. The outcome of toxicity was categorized into three classes: complete renal recovery in absence of AKI criteria (i.e., last serum creatinine < 1.5-fold baseline); no recovery if patients needed RRT, and partial recovery if, at the last evaluation, patients were not on RRT but failed to meet the criteria for complete renal recovery (54).

Finally, when present we consider the tumor radiological response that was reported as: complete response, partial response, and stable disease.

### Statistical analysis

Descriptive and statistical analyses were performed on the whole of our center’s cases and those extracted from the literature.

Quantitative variables were represented by mean ± standard deviation (SD) or interquartile ranges (IQR) if they were not normally distributed (Shapiro Test).

Group analyses for continuous variables were performed by using the Student t-test or nonparametric Mann-Whitney test when appropriate. Comparisons of proportions were made using Fisher’s exact test. Univariate and multivariate logistic regression analysis was used to investigate predictor factors on AKI severity and renal function recovery.

The analyses were performed using Stata 13.1 (Stata Corporation, College Station, Tex, United States) used for computation. A 2-sided P value <0.05 was considered statistically significant.

## Results

### Patient characteristics

Eighty-five patients (56 males) with ICI-related biopsy-proven ATIN were analyzed. The mean age was 61.4 ± 19 years, and the most frequent comorbidities were hypertension (n=41), ischemic heart disease/atrial fibrillation (n=12), diabetes mellitus (n=8), and hypothyroidism (n=7). Forty-three patients (51%) had melanoma, 25 (30%) had non-small cell lung cancer (NSCLC), 8 had clear cell renal cell carcinoma (CRCC), and 9 had other types of cancer. ICI treatment consisted of PD-1 inhibitors nivolumab in 28 patients, pembrolizumab in 21 patients, PDL-1 inhibitor (atezolizumab) in 2 patients, and CTLA4 inhibitor (ipilimumab) in 9 patients, while 19 patients were treated with dual ICI blockade, nivolumab+ipilimumab. In six cases, the checkpoint inhibitor drug was not specified (**Table 1**). Eleven patients in the whole group were receiving chemotherapy combined with immunotherapy.

**Table 1:** Clinical and laboratory characteristics of patients evaluated in the pooled analysis.

**N patients**	85
**Age, years**	61.4 ± 19
**Sex-m (%)**	56 (65.9)
	N (%)
Comorbidities, n	61
- **Hypertension**	41 (67)
- **Heart disease**	12 (19.6)
- **Diabetes**	8 (13)
Cancer, n	85
**-** **Melanoma**	43 (50.6)
**-** **NSCLC**	25 (29.4)
**-** **CRCC**	8 (9.4)
**-** **Other**	9 (10.6)
ICI drug, n	85
**-** **Nivolumab single agent**	28 (33)
**-** **Pembrolizumab**	21 (24.7)
**-** **Atezolizumab**	2 (2.3)
**-** **Ipilimumab single agent**	9 (10.5)
**-** **Nivolumab+Ipilimumab**	19 (22.5)
**-** **Unspecified**	6 (7)
Therapy line	
**-** **First line (n=49)**	27 (55)
**Therapy cycles at AKI, median (IQR)**	4 (2.2-6.7)
Concomitant medications,	
**-** **PPIs (n=69)**	42 (60.8)
**-** **NSAIDs (n=65)**	8 (12.3)
**-** **RAASi (n=63)**	12 (19)
Renal outcomes, n	80
**-** **Complete recovery**	32 (40)
**-** **Partial recovery**	45 (56.2)
**-** **No recovery**	3 (3.8)

Percentages were expressed considering the data available for each parameter.

ICI, immune checkpoint inhibitors; NSCLC, non-small cell lung cancer; CRCC, clear cell renal cell carcinoma; PPIs, proton pump inhibitors; NSAIDs, nonsteroidal anti-inflammatory drugs; RAASi, renin-angiotensin-aldosterone system inhibitors.

### Clinical presentation and ICI-related renal toxicity

The mean basal serum creatinine (available in 78 patients) was 0.8 ± 0.4 mg/dl.

Renal toxicity developed during the first line in 27 patients out of the 49 for whom data were available (55%). Instead, data on therapy cycles were present in 62 cases showing that AKI developed after a median of four cycles of therapy, but in most cases (n=48, 77%) after at least three treatment cycles. Eleven patients (14%) presented with AKI stage 1, 16 patients (20.5%) with stage 2, and 51 patients (65.5%) with AKI stage 3, including five patients requiring RRT. In 7 patients AKI stage was not reported, and it was not possible to evaluate the stage due to the lack of data on basal serum creatinine. Urinary sediment, available in 55 patients, showed: 36 patients with leukocyturia, 20 patients with hematuria, and 13 patients with casts. Five patients (9%) presented with nephrotic range proteinuria, and ten patients (18%) had gross hematuria. Data on the clinical presentation of renal toxicity were available for 41 patients, among them 7 presented with edema, 10 had fever, and 12 were hypertensive. AKI was accompanied by other ICI-related toxicity in 37/74 patients (50%), including 11 cases of autoimmune endocrinological disorders, 8 cases of skin toxicity, and 5 cases of autoimmune hepatitis. Due to the study design, kidney biopsy showed a histological picture of ATIN in all the patients (**Supplementary Figure 2**). According to the report of histological data, in 12 out of 61 patients (20%), interstitial granulomas were described, while ATN was present in 10 out of 71 patients (14%). Comparing patients with the more severe form of AKI (AKI 3) with those presenting milder renal dysfunction (AKI 1-2), we found no significant difference in patient demographic and clinical characteristics, such as in histological findings (**Table 2**).

**Table 2:** Clinical and laboratory characteristics of patients with ICI-related ATIN according to the acute kidney injury severity.

	AKI 1-2 (n=27)		AKI 3 (n=51)		p
	N data available		N data available		
**Age, years**	27	56.9± 24.4	51	64.2± 15.3	0.6
**Basal creatinine, mg/dl**	27	0.97± 0.18	51	0.98± 0.4	0.4
**Therapy course (n)**	21	7.6 ± 7.5	44	6.8± 6.7	0.58
		n (%)		n (%)	
**Male**	27	14 (51.9)	51	37 (72.5)	0.08
**Hypertension**	23	14 (56)	44	26 (59)	0.9
**Diabetes**	23	3 (15)	44	5 (11)	0.9
**PD-1i+CTLA4i (n 19) **		0 (0)		19 (100)	<0.001
**Single ICI agent (n 60)**		31 (52)		29 (48)	
**Concomitant Chemotherapy**	23	3 (13.0)	49	8 (16.3)	0.8
**First therapy course**	20	8 (40.0)	24	17 (70.8)	0.039
Histological findings					
**Granulomas**	15	1 (6.7)	42	10 (23.8)	0.1
**ATN**	21	3 (14.3)	43	5 (11.6)	0.7
Concomitant medications					
**PPIs**	27	17 (63)	42	24 (57.1)	0.5
**NSAIDs**	25	3 (12.0)	39	4 (10.3)	
**Other ICI-related toxicity**	27	12 (44.4)	40	21 (52.5)	0.5
Renal outcomes	27		50		
**Complete recovery**		15 (55.6)		15 (30)	0.04
**Partial recovery**		12 (44.4)		32 (64)	
**No recovery**		0 (0)		3 (6)	

ICI, immune checkpoint inhibitors; ATIN, acute tubulointerstitial nephritis; AKI, acute kidney injury; ATN, acute tubular necrosis; PD-1, programmed Death protein 1; CTLA-4, cytotoxic T Lymphocyte Antigen 4; PPIs, proton pump inhibitors; NSAIDs, nonsteroidal anti-inflammatory drugs.

*In 7 patients AKI stage was not reported. Moreover, it was not possible to evaluate the stage due to the lack of data on basal serum creatinine.

**Figure 1:**
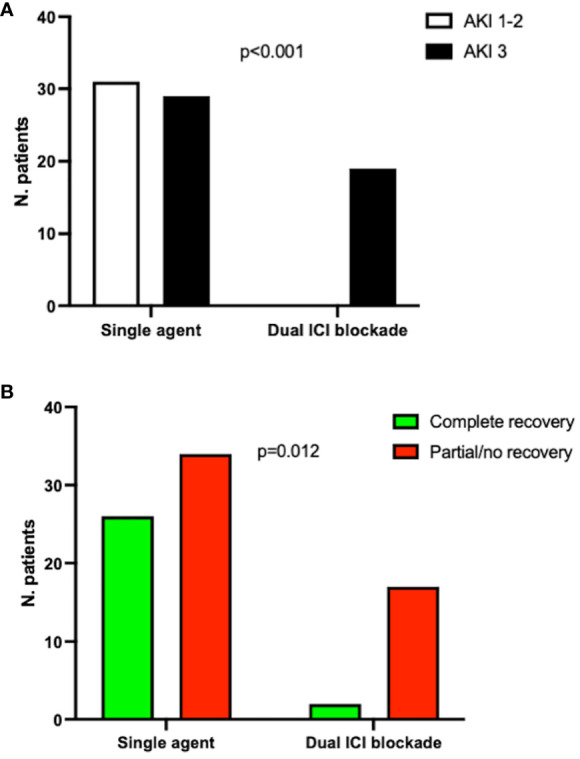
Prevalence of AKI stage 3 **(A)** and renal recovery (complete vs partial/no recovery) **(B)** in patients on single or dual ICI blockade. Fisher’s test. ICI, immune checkpoint inhibitors; AKI, acute kidney injury. Six patients were not included in this analysis due to the lack of data on specific ICI treatment prescribed.

Among AKI 3 patients, there was a significantly higher prevalence of patients at the first-line therapy (p=0.04). All the patients treated with the dual ICI blockade developed stage 3 AKI (100%), compared with 29 out of the 60 patients (48%) taking a single agent (p<0.001, **Figure 1A**).

Moreover, patients developing AKI stage 3 presented worse renal outcomes in terms of a significantly higher number of patients experiencing partial or no recovery of renal function.

Multivariate logistic regression analysis evaluating the risk of developing AKI stage 3 showed that when combined ICI treatment was considered in the analysis, other factors, such as age, sex, and therapy line lost significance (**Supplementary Table 1**).

### Treatment of ICI-related ATIN

Data on ATIN treatment were reported in 84 patients. Among them, seventy-seven patients (91%) received immunosuppressive treatment, while seven patients did not receive any specific therapy.

All the treated patients received corticosteroids at different dosages, associated with mycophenolate mofetil in 5 patients and Rituximab in 1 case.

Excluding one patient still taking MMF three years after the onset of nivolumab-related toxicity, the mean duration of immunosuppressive treatment was 68.3 ± 49.3 days.

ICI treatment was withdrawn in 65 out of 69 patients with available data. Following renal function improvement, in 15 patients ICI therapy was restarted, and in six patients (40%) AKI recurred.

### Renal outcomes

Renal outcomes were reported in 80 patients. Overall, at the last control (time ranging 7-1400 days after AKI diagnosis), 32 patients (40%) presented a complete renal recovery, 45 patients (56.2%) had a partial recovery, and 3 patients (3.8%) did not recover.

Complete renal recovery was observed only in 2 out of the 7 patients (28%) not treated with steroids, compared with 30 out of 77 treated patients (39%, p=ns).

Then, we found that among patients without complete recovery, there was a higher prevalence of dual ICI therapy and AKI stage 3 at the presentation (p=0.002 and 0.026, respectively), with no significant difference in other clinical characteristics (**Table 3**).

**Table 3:** Clinical and laboratory characteristics of patients with ICI-related ATIN according to the renal function recovery.

	Complete recovery (n=32)		Partial/no recovery (n=48)		p
	N data available		N data available		
**Age (years)**	32	59.2± 22.5	48	63.7± 16	0.7
**Baseline creatinine, mg/dl**	32	0.9 ± 0.3	48	0.8± 0.46	0.08
**Therapy course (n)**	27	4 (2-10)	39	5 (3-8)	0.8
		n (%)		n (%)	
**Male**	32	21 (65.6)	48	33 (68.8)	0.8
**Hypertension**	25	20 (80)	35	21(60)	0.15
**Diabetes**	25	4 (16)	35	4 (11.5)	0.7
**PD-1i+CTLA4i**	30	2 (6.6)	44	17 (38)	0.002
**Concomitant Chemotherapy**	30	6 (20.0)	44	5 (11.4)	0.33
**First therapy course**	27	13 (48.1)	18	12 (27.3)	0.6
**AKI stage 3**	30	15 (50.0)	46	35 (76.0)	0.026
**Other ICI-related toxicity**	32	17 (53.1)	37	18 (48.6)	0.9
**Proteinuria**	22	16 (72.7)	34	19 (51.4	0.26
**Hematuria**	22	7 (31.8)	39	5 (13.9)	0.08
**Corticosteroid treatment**	32	30 (93.8)	47	42 (89.4)	0.7
**Immunosuppression duration (days)**	16	61.4 ± 52	14	76.2 ± 46	0.12

ICI, immune checkpoint inhibitors; ATIN, acute tubulointerstitial nephritis; AKI, acute kidney injury; PD-1, programmed Death protein 1; CTLA-4, cytotoxic T Lymphocyte Antigen 4.

Conversely, a significantly higher percentage of patients taking a single ICI agent, 26 out of 60 (43%), had a complete renal recovery when compared with patients taking dual therapy (2 out of 19 (10%), p=0.01, **Figure 1B**). At univariate logistic regression, complete renal recovery was inversely associated with dual ICI blockade (OR 0.1, 95CI 0.02-0.5, p=0.006) and AKI 3 (OR 0.31, 95CI 0.1-0.9, p=0.04), but only the association with dual ICI therapy remained significant at multivariate analysis (**Supplementary Table 2**).

Finally, tumor radiological response was reported only in 33 cases, showing a complete response in seven patients (21.5%), a partial response in 10 patients (30%) and stable disease in the remaining 16 patients (48.5%).

## Discussion

In this paper, we provide information about the risk profile, clinical presentation, and management of AKI due to ATIN occurring during ICIs. The rationale of this study moved from the observation that most of the previous reports on AKI during ICIs did not distinguish the various potential underlying causes, and there is limited data on patients undergoing kidney biopsies. This may be a relevant weakness since AKI in cancer patients may recognize multiple causes, including irAEs, volume depletion, obstructive nephropathy, hypercalcemia, and drug-related nephrotoxicity (55).

So, for example, only a part (151/429- 35%) of the large cohort of patients with ICI-related AKI, reported by Gupta et al., underwent kidney biopsy and, among them, almost 20% of the patients presented other nephropathies than ATIN (52).

So, conceivably the authors analyzed a heterogenic population, including an unknown number of patients without ATIN, which may explain some discrepancies we found comparing their results with our data. For example, Gupta et al. described a lower percentage of patients with AKI stage 3 (48.5 vs. 65.5% of our study). These differences may be at least in part influenced by different populations analyzed, further highlighting that proper discrimination of AKI etiologies is essential to define disease course and may have important clinical implications.

This is why, we focused our attention on cases of biopsy-proven ATIN, the most common histological presentation of renal irAEs during ICIs. This analysis allowed us to make many considerations that could help in guiding clinical decisions and future research.

First, we found that the timing of AKI development may be noteworthy. Indeed, as partially already reported, we observed that ATIN may develop also late in the history of cancer therapy, after several therapy cycles (56).

So, caution and awareness for the development of renal toxicity should be maintained throughout the whole course of therapy. Notably, it seems that dual ICI blockade with combined anti-PD-1 and anti-CTLA-4 treatment is associated with a significant risk of severe AKI and a reduced chance of renal recovery. This occurrence is not unexpected, as it is known that dual blockade is associated with an increased risk of irAEs compared to single-agent ICI (57). The potential elevated harmful effects of combination therapy may also explain the observation of an increased AKI severity in patients undergoing ICIs as first-line therapy. Indeed, combinations are typically administered at the first line, while patients treated in the second or further line usually receive single-agent PD-1/PD-L1 inhibitors (58). However, due to the limited number of cases, our data did not allow us to discriminate the nephrotoxic effects of the different ICI classes (CTLA4, PD-1, and PD-L1 inhibitors).

Almost all the patients included in this analysis were treated with corticosteroids, so we cannot quantify the impact of this treatment on the recovery of renal function in our cohort. However, as a matter of fact, corticosteroids are the cornerstone therapy in cases of ATIN of different etiologies (59). Moreover, general observations involving irAEs and immune-related renal impairment during ICIs confirm the effectiveness of corticosteroids (60). So, conceivably, a prompt corticosteroid initiation at the onset of renal toxicity might be instrumental to the resolution of the event, and this approach is not generally considered to disrupt the anti-neoplastic activity of ICIs. On the other hand, this is a fundamental point in favor of the renal biopsy. Indeed, apart from ATIN, other structural renal alterations may be found, which deserve different treatments and have various prognosis (61). Moreover, renal biopsy may guide differential diagnosis even when multiple drug-related nephrotoxicities are suspected (62) Finally, renal biopsy is important also to provide information on irAE severity and prognosis, which may assist in the following clinical management of the patient, such as the decision on ICI rechallenging (63).

Notably, our suggestions are not completely in line with the current recommendations from the American Society of Clinical Oncology (ASCO), which, although emphasizes the use of steroids for the treatment of suspected ICI-related AKI, suggest performing a kidney biopsy only in patients with severe AKI-stage 3 (64). Indeed, for the reasons above exposed we think that a kidney biopsy should be considered in almost all AKI patients fit for the procedure.

Looking at renal outcomes, we observed that in many patients renal recovery was not complete. This is an extremely relevant observation because permanent renal dysfunction in the case of patients with advanced cancer often results in further deterioration of quality of life and possibly a reduction of survival due to the worsened clinical condition and inability to receive further antineoplastic treatments. On the other hand, patients with potentially curable cancer could present the risk of developing chronic kidney disease (65). Finally, a further observation deserving of attention is the high recurrence rate of AKI after ICI reuse, which may have possible relevant clinical implications.

Remarkably, even in the analysis of the renal outcomes, we noticed significant differences in our patients when compared with those studied by Gupta et al. (52) In particular, they found better outcomes, with a higher rate of renal recovery and a lower rate of recurrence. Also, in this case, these dissimilarities could have been partially explained by the different populations evaluated, even if they could have been conditioned by the limitation of our study. Indeed, we are aware that the study has some weaknesses, mainly due to its retrospective design and collection of pooled data from the literature. So, although we sought uniform data collection and definitions, case reports from different authors usually lack the consistency of systematic data collection and may be biased from different diagnostic and clinical approaches (66). Moreover, our data are not representative of the general population of patients treated with ICIs, since, in this study, we intended to study the specific condition of ATIN. Furthermore, since there is a great heterogeneity in kidney biopsy policy among different centers, it is not possible to rule out that a selection bias occurred, mainly due to mild-moderate AKI cases who did not undergo kidney biopsy.

Finally, due to the lack of a control group and complete clinical and laboratory data, we could not evaluate the prevalence of other known risk factors for AKI, such as concomitant medications, hyperuricemia, and anemia, that may impact the susceptibility of developing kidney damage (67).

Surely, the evaluation of AKI timing, adverse effects of dual ICI blockade, and the long-term outcome of ATIN, such as the establishment of standardized therapeutic approaches, need prospective studies to be elucidated. Nevertheless, generating such data might be difficult due to the need for focused analysis including large cohorts of patients undergoing renal biopsy for AKI.

## Conclusions

In conclusion, we found that ICI-related ATIN may develop at any stage of therapy in patients treated with ICIs. It may present as a severe form of AKI, particularly in patients with dual ICI blockade. This condition appears partially reversible, but concerns remain about the renal function sequelae and the possibility of restarting treatment after AKI resolution due to the risk of recurrence. Thus, we suggest that a simple but complete renal risk assessment should be included in the workup of patients undergoing ICI therapy, especially if a combined regimen is prescribed (68, 69). At the same time, monitoring renal function should be warranted throughout the whole course of immunotherapy, pointing out the fundamental contribution of renal biopsy in proper clinical management.

## Data availability statement

The raw data supporting the conclusions of this article will be made available by the authors, without undue reservation.

## Author contributions

PE, AB, CG contributed to conception and design of the study. AB, EL, MP organized the database. FC, LM performed the statistical analysis. PE wrote the first draft of the manuscript. CG, FV wrote sections of the manuscript. All authors contributed to the article and approved the submitted version.
