# Supplementary material for: Biopsy-proven acute tubulointerstitial nephritis in patients treated with immune checkpoint inhibitors: a pooled analysis of case reports
Source: Front Oncol. 2023 Oct 23;13:1221135. doi: 10.3389/fonc.2023.1221135 (PMC10627243; doi:10.3389/fonc.2023.1221135)
Supplement: Supplementary file 1 [file DataSheet_1.docx]

Supplementary Material

**Biopsy-proven acute tubulointerstitial nephritis in patients treated with immune checkpoint inhibitors: A pooled analysis of case reports**

**Pasquale Esposito ^1,2^, Annarita Bottini ^2^, Elvina Lecini ^2^, Francesca Cappadona ^1^, Michela Piaggio^2^, Lucia Macciò ^2^, Carlo Genova ^2,3^, Francesca Viazzi ^1,2^.**

*** Correspondence:**Pasquale Esposito,

Unit of Nephrology, Dialysis and Transplantation, Department of Internal Medicine, University of Genoa and IRCCS Ospedale Policlinico San Martino, Genova, Italy

e-mail address: [Pasquale.esposito@unige.it](mailto:Pasquale.esposito@unige.it)

**SUPPLEMENTARY TABLE**

**Supplementary Table 1. Logistic regression for the risk of developing AKI stage 3 in patients with ICI-related ATIN**

|  | **Univariate** |  |  | **Multivariate** |  |  |
| --- | --- | --- | --- | --- | --- | --- |
|  | *OR* | *95CI* | *p* | *OR* | *95CI* | *p* |
| **PDi+CTLA4i*** | 1 |  |  | 1 |  |  |
| **First therapy course** | 3.6 | 1.03-12.7 | 0.043 | 2.8 | 0.74-10.6 | 0.12 |
| **Age** | 1.01 | 0.99-1 | 0.11 | 1.01 | 0.98-1.05 | 0.42 |
| **Sex (female)** | 0.4 | 0.15-1 | 0.07 | 1.28 | 0.3-5 | 0.7 |

Abbreviations: immune checkpoint inhibitors (ICI), acute tubulointerstitial nephritis (ATIN), acute kidney injury (AKI), programmed Death protein 1 (PD-1), cytotoxic T Lymphocyte Antigen 4 (CTLA-4).

*all the patients undergoing combined ICI-treatment developed AKI stage 3

**Supplementary Table 2 Logistic regression for the complete renal recovery in patients with ICI-related ATIN**

|  | **Univariate** |  |  | **Multivariate** |  |  |
| --- | --- | --- | --- | --- | --- | --- |
|  | *OR* | *95CI* | *p* | *OR* | *95CI* | *p* |
| **PDi+CTLA4i** | 0.1 | 0.02-0.5 | 0.006 | 0.16 | 0.03-0.8 | 0.034 |
| **AKI stage 3** | 0.31 | 0.11-0.84 | 0.021 | 0.4 | 0.1-1.3 | 0.1 |
| **Age** | 0.98 | 0.96-1.01 | 0.3 | 1 | 0.9-1.02 | 0.87 |
| **Sex (female)** | 1.15 | 0.45-2.9 | 0.77 | 0.93 | 0.3-2.9 | 0.9 |

Abbreviations: immune checkpoint inhibitors (ICI), acute tubulointerstitial nephritis (ATIN), acute kidney injury (AKI), programmed Death protein 1 (PD-1), cytotoxic T Lymphocyte Antigen 4 (CTLA-4).

**SUPPLEMENTARY FIGURES**

**
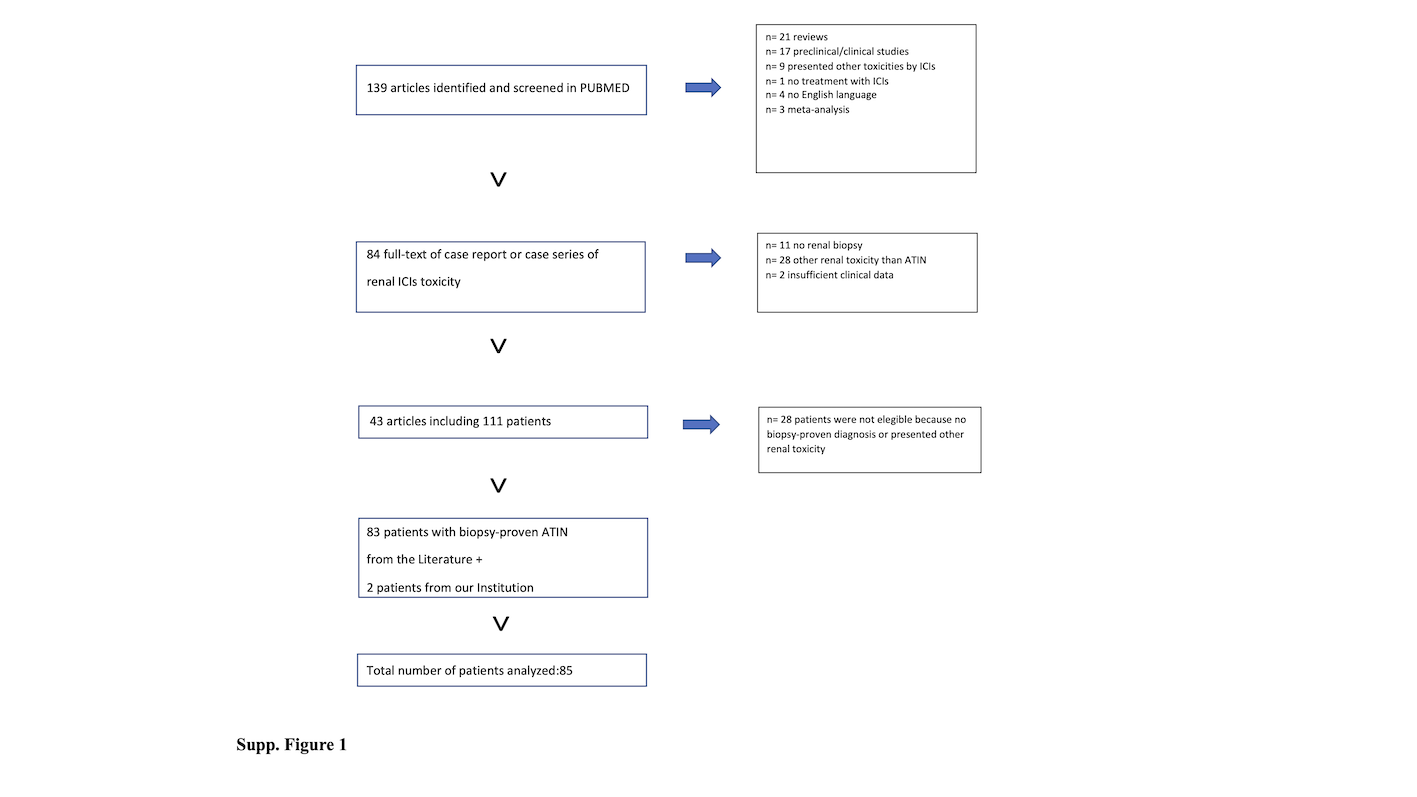
**

**Suppl Figure 1.** Selection strategy of cases reports included in the pooled analysis.

Abbreviations: immune checkpoint inhibitors (ICI), acute tubulointerstitial nephritis (ATIN)

**
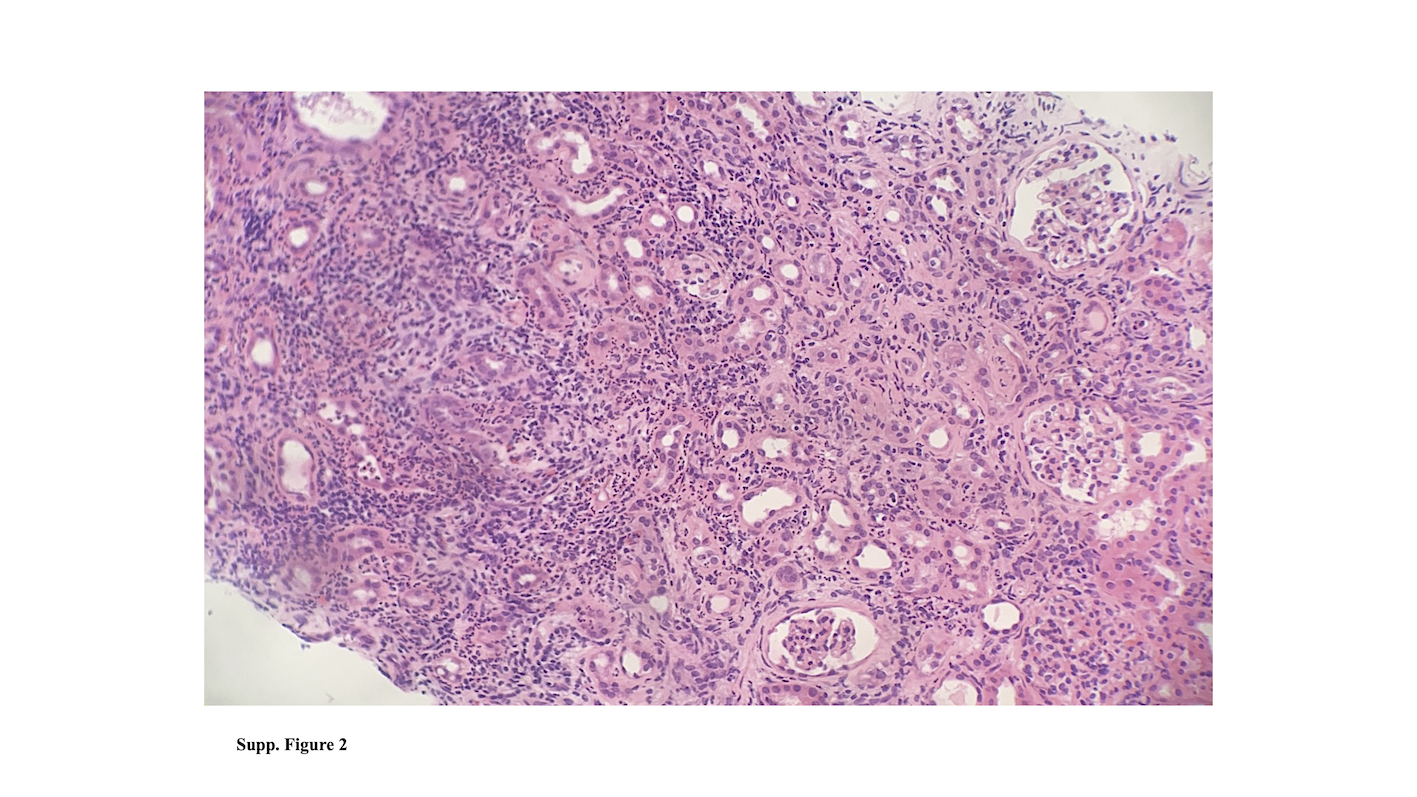
Suppl Figure 2.** Exemplificative kidney biopsy findings in a patient with ICI-related ATIN: interstitial nephritis with severe interstitial inflammation including neutrophils, plasmacells and mononuclear cells; tubulitis is also present. H&E 40x. The biopsy is from a patient diagnosed at our Institution, who was included in the study.

Reprinted by permission from Springer Nature; Picciotto, D. *et al.* (2022). An Updated Focus on Immune Checkpoint Inhibitors and Tubulointerstitial Nephritis. In: Interdisciplinary Cancer Research. Springer, Cham.
